# Development of a Screening Algorithm for Alzheimer's Disease Using Categorical Verbal Fluency

**DOI:** 10.1371/journal.pone.0084111

**Published:** 2014-01-02

**Authors:** Yeon Kyung Chi, Ji Won Han, Hyeon Jeong, Jae Young Park, Tae Hui Kim, Jung Jae Lee, Seok Bum Lee, Joon Hyuk Park, Jong Chul Yoon, Jeong Lan Kim, Seung-Ho Ryu, Jin Hyeong Jhoo, Dong Young Lee, Ki Woong Kim

**Affiliations:** 1 Department of Neuropsychiatry, Seoul National University Bundang Hospital, Seongnam, Korea; 2 Department of Psychiatry, Kyungbook National University Hospital, Daegu, Korea; 3 Department of Psychiatry, Dankook University Hospital, Cheonan, Korea; 4 Department of Neuropsychiatry, Jeju National University Hospital, Jeju, Korea; 5 Department of Neuropsychiatry, Kyunggi Provincial Hospital for the Elderly, Yongin, Korea; 6 Department of Psychiatry, Chungnam National University Hospital, Daejeon, Korea; 7 Department of Neuropsychiatry, Konkuk University Hospital, Seoul, Korea; 8 Department of Neuropsychiatry, Kangwon National University Hospital, Chooncheon, Korea; 9 Department of Neuropsychiatry, Seoul National University Hospital, Seoul, Korea; 10 Department of Psychiatry, Seoul National University College of Medicine, Seoul, Korea; 11 Department of Brain and Cognitive Sciences, Seoul National University College of Natural Sciences, Seoul, Korea; University of Nebraska Medical Center, United States of America

## Abstract

We developed a weighted composite score of the categorical verbal fluency test (CVFT) that can more easily and widely screen Alzheimer's disease (AD) than the mini-mental status examination (MMSE). We administered the CVFT using animal category and MMSE to 423 community-dwelling mild probable AD patients and their age- and gender-matched cognitively normal controls. To enhance the diagnostic accuracy for AD of the CVFT, we obtained a weighted composite score from subindex scores of the CVFT using a logistic regression model: logit (case)  = 1.160+0.474× gender +0.003× age +0.226× education level – 0.089× first-half score – 0.516× switching score -0.303× clustering score +0.534× perseveration score. The area under the receiver operating curve (AUC) for AD of this composite score AD was 0.903 (95% CI = 0.883 – 0.923), and was larger than that of the age-, gender- and education-adjusted total score of the CVFT (p<0.001). In 100 bootstrapped re-samples, the composite score consistently showed better diagnostic accuracy, sensitivity and specificity for AD than the total score. Although AUC for AD of the CVFT composite score was slightly smaller than that of the MMSE (0.930, p = 0.006), the CVFT composite score may be a good alternative to the MMSE for screening AD since it is much briefer, cheaper, and more easily applicable over phone or internet than the MMSE.

## Introduction

With the world population aging, the number of dementia patients worldwide will be increasing rapidly. The number of dementia patients is projected to double every 20 years to 2.1 million and the national cost of dementia every 10 years to 73.8 billion USD by 2050 in South Korea [1], [2]. Alzheimer's disease (AD) approximately accounts for two-thirds of overall dementia, making AD the most prevalent form of dementia [3]. As more interventions for AD become available, there is an increasing need for screening tests that can accurately detect early-stage AD.

Ideally, a screening test for AD should be sensitive and specific enough to identify cognitively impaired individuals who need further comprehensive evaluation and management. Simultaneously, a range of health care personnel should be able to quickly and easily administer it. It would be so much the better the test can be self-administered without assistance from health care personnel.

The mini–mental state examination (MMSE) has been used most widely to screen for dementia in both clinical and research settings [4]. However, the MMSE has several limitations as a screening instrument for AD. First, it cannot be administered to the disabled elderly with motor or vision impairments because it involves naming ability, visuospatial ability, and praxis. Second, it is not brief enough since it takes about 15 minutes. Third, its items for measuring memory are limited (6 points). Fourth, it is insensitive to frontal dysfunction, which is often present in early AD [5].

In this sense, the categorical verbal fluency test (CVFT) can be an attractive alternative of the MMSE for screening AD in several ways. First of all, the CVFT is much shorter and easier to administer than the MMSE. It takes about only 3 minutes and requires minimal training to administer. Second, it measures frontal function as well as memory. Both episodic and semantic memories are impaired in early stage of AD, and word finding difficulty is common in early stage of AD [6], [7]. Third, it can be applicable much more widely than the MMSE since it does not require motor performance or vision.

However, its diagnostic accuracy for AD is a major limitation as a screening instrument for AD. The total score (total number of correct words produced in a given time interval) of CVFT, a conventional index of it, is much less accurate in diagnosing AD than the MMSE since the total score of CVFT is neither specific to temporal lobe dysfunction nor sensitive to diffuse brain damage [8]. To overcome this limitation of the CVFT, we should integrate its sub-indices that may be sensitive to memory impairment and/or executive dysfunctions into a new index that may have an acceptable diagnostic accuracy for AD.

The CVFT has several sub-indices. Clustering is the production of words within semantic subcategories, and switching is the ability to shift between clusters. Clustering relies on temporal lobe processes that are related to the integrity of the lexico-semantic network, and switching involves frontal lobe processes associated with strategic search and retrieval [8]–[10]. Perseveration errors are repetitive productions of words that have already been produced in the sequence, and intrusion errors are insertions of words that do not belong to the required semantic category [11], [12]. Participants must monitor the output and in particular, suppress previously recalled items to avoid perseveration errors. Perseveration errors are associated with the capacity of working memory [13], [14]. Intrusion errors depend on the size of the semantic field to be explored. AD patients, in particular, were more likely to show perseveration errors in CVFT than normal controls [13], [14]. First-half and second-half scores can reflect the decline of word production over time in a given time interval (typically one minute) [12], [15], [16]. Word production in the first-half (0–30 seconds) mainly depends on clustering whereas that in second-half (31–60 seconds) depends on both clustering and switching [12]. In the first-half interval, word production relies on the ability to rapidly access words that are readily available from semantic memory. During the second-half interval, word production requires more effort and is dependent on semantic network organization [12] since the readily-accessible word-pool is exhausted [16].

In the present study, we developed a new composite score of the CVFT using these six subindices and demographic characteristics and examined its diagnostic accuracy for AD.

## Methods

### Participants

This study was a retrospective study using the data of probable AD patients enrolled from both communities and clinics. We selected the patients from both the participants in the Korean Longitudinal Study on Health and Aging (KLOSHA) [17] or the Nationwide Survey on Dementia Epidemiology of Korea (NaSDEK) [3] and the visitors to the Dementia Clinics of 5 university hospitals (Seoul National University Bundang Hospital, Chungnam National University Hospital, Kyungbook National University Hospital, Jeju National University Hospital, Dankook University Hospital) from 2007 to 2011. Among the 1,023 probable AD patients, we select 423 patients only whose Clinical Dementia Rating (CDR) [18] was 0.5 or 1, the MMSE was 13 or higher, and age- and gender-matched cognitive normal controls subject was available. We selected the 423 age- and gender-matched control subjects from the participants of the KLOSHA or the NaSDEK. All control subjects had the CDR of 0.

All subjects were community-dwelling Koreans aged 60 years or more who had adequate vision and hearing, although many wore glasses, and some required a hearing aid. The subjects who had major or minor depressive disorders, other Axis I psychiatric disorders and serious medical or neurological disorders that could affect their cognitive function were excluded. The subjects whose scores of the Korean version of Geriatric Depression Scale (GDS) or its short form were higher than the cut-off scores of clinically significant depression were also excluded [19].

All subjects were fully informed of the study protocol. The subjects themselves or their legal guardians provided written informed consent.

### Assessments

Geropsychiatrists with advanced training in neuropsychiatry and dementia research examined all subjects including according to the protocol of the Korean version of the Consortium to Establish a Registry for Alzheimer's Disease (CERAD-K) Clinical Assessment Battery (CERAD-K-C) [20]. We interviewed reliable informants of the AD patients to acquire accurate information regarding subjects' cognitive and functional changes and medical histories. A panel of 4 research geropsychiatrists determined diagnoses and CDR. CDR of 0.5, 1, 2, and 3 indicate very mild, mild, moderate and severe degree of dementia, respectively. We diagnosed probable AD according to the criteria of the National Institute of Neurological and Communicative Disorders and Stroke and the Alzheimer's Disease and Related Disorders Association (NINCDS-ADRDA) [21].

Psychologists or trained research nurses administered the CVFT and the Korean version of MMSE (MMSE) to each subject. In conducting the CVFT, we instructed each participant to generate names of animals for 60 seconds. We obtained 7 indices from the responses of each participant: (1) the total score, which was the number of overall correct responses generated within 60 seconds; (2) the first-half score, which was the number of correct responses generated within the first 30 seconds; (3) the second-half score, which was the number of correct response generated during the last 30 seconds; (4) the perseveration score, which was the number of repetitive correct or incorrect responses; (5) the intrusion score, which was the number of non-animal responses; (6) the clustering score, which was the mean cluster size; and (7) the switching score which was the number of switches between clusters. A cluster was defined as a group of successively generated words belonging to the same subcategory such as farm animals, pets, Asian animals, African animals, various zoological categories (e.g., birds, felines, fish), and Chinese zodiac animals. The cluster size was defined as the number of correct responses belonging to each subcategory minus 1. Details of the rules we followed for assessing scores for switching and clustering are published in according to Troyer et al [9].

This study was conducted in accordance with the latest version of the Declaration of Helsinki. The details of the study protocol were reviewed and approved by the institutional review board of Seoul National University Bundang Hospital.

### Development of the composite score

We developed a weighted composite score (CVFT-C) from the six subindex scores using stepwise and forward selection methods of logistic regression analysis. These analyses were adjusted for age, gender, and educational level. We combined the best subset method with the Akaike Information Criterion (AIC) to make the best use of information and to avoid over-fitting. Then a weighted composite score was calculated using the logit of subindex scores weighted by their coefficients from logistic regression models.

### Statistical analyses

Descriptive statistics were used to determine whether differences in the demographic and clinical characteristics of the subjects existed. Student's *t* tests and chi square tests were used to compare demographics of the AD group and the control group. Multivariate analyses of covariance (MANCOVA), adjusted for subject education level, were used to compare the MMSE total scores and CVFT scores between the AD and control groups.

We compared the diagnostic accuracy for AD of the CVFT-C with those of age-, gender- and education-adjusted CVFT total scores (CVFT-T) and age-, gender- and education-adjusted MMSE score (MMSE-T). We determined diagnostic accuracy by comparing the areas under the receiver operating characteristics (ROC) curve (AUC), and compared the AUC between the CVFT-C, CVFT-T and MMSE-T using the method proposed by Hanley and McNeil [22].

Then, we estimated confidence intervals of the AUC, sensitivity, and specificity for AD using a simple bootstrap procedure (100 resamples). In each run, 60% of samples were randomly selected. In this procedure, the patient data set was repeatedly divided through random sampling into a training set to derive a composite index through logistic regression and a test set for computing sensitivities and specificities. The results from multiple runs were then aggregated to form the bootstrap estimate of sensitivity and specificity. Statistical analyses were performed using SAS ver. 9.2 (SAS institute, Inc., Cary, NC, USA), SPSS ver. 18 (SPSS inc., Chicago, IL USA), and MedCalc ver. 8.10.0 (Medcalc softwear, Mariakerke, Belgium). P values of less than 0.05 were indicated statistically significant results.

## Results

Table 1 shows the characteristics of the participants. The MMSE and CVFT total score of the AD group were lower than those in the control group (p<.001). Among the 6 index scores of the CVFT, the first-half, second-half and switching scores were lower in the AD group than in the control group (p<.001). The clustering (p = .733), perseveration (p = .078), and intrusion (p = .808) scores were comparable between the two groups.

**Table 1:** Comparison of Mini Mental Status Examination (MMSE) and Categorical Verbal Fluency Test (CVFT) scores between normal controls and Alzheimer's disease (AD) patients.

Variable	Normal controls	AD patients	Significance
Numbers of subjects	423	423	-
Age (years, mean ± SD)	75.68±6.5	75.68±6.5	
Education (years, mean ± SD)	2.86±3.6	6.1±5.2	<0.001^a^
Gender (women, %)	66.4	66.4	<0.001^b^
MMSE (points, mean ± SD)	23.3±3.7	18.1±3.9	<0.001^c^
CVFT (points, mean ± SD)			
Total score	12.2±4.0	8.9±3.6	<0.001^c^
First-half score	8.5±2.6	6.5±2.6	<0.001^c^
Second-half score	3.7±2.3	2.4±2.7	<0.001^c^
Switching score	10.39±8.0	2.4±2.2	<0.001^c^
Clustering score	2.8±2.2	2.6±2.1	0.733^c^
Perseveration score	0.67±1.2	0.94±1.5	0.078^c^
Intrusion score	0.07±0.5	0.05±0.5	0.808^c^

^a^ Student's *t*- test.

^b^ Pearson Chi-square test.

^c^ Multivariate analysis of covariance, adjusting for education.

In order to investigate the optimal regression model to discriminate the AD group from the control group, we performed a forward stepwise logistic regression analysis, adjusting for age, gender, and education level. As shown in Table 2, the scores for first-half, switching, clustering and perseveration were included in the final model. We used the equation as following to calculate the weighted composite score from the final logistic regression model for discriminating individuals with AD from normal controls; *logit (case)  = 1.160 +0.474× gender +0.003× age +0.226× education level – 0.089× first-half score – 0.516× switching score – 0.303× clustering score +0.534× perseveration score*.

**Table 2:** Logistic regression model for predicting Alzheimer's disease using the Categorical Verbal Fluency Test (CVFT).

	B (SE)	Wald	Significance	OR (95% CI)
Demographic				
Gender	0.474 (0.23)	4.28	0.039	1.607 (1.025–2.519)
Age	0.003 (0.02)	0.05	0.826	1.003 (0.974–1.033)
Education level	0.226 (0.03)	70.06	<0.0001	1.254 (1.189–1.322)
CVFT				
Perseveration score	0.534 (0.10)	29.78	<0.0001	1.705 (1.408–2.065)
First-half score	−0.089 (0.05)	3.96	0.047	0.915 (0.838–0.999)
Switching score	−0.516 (0.06)	74.23	<0.0001	0.597 (0.531–0.671)
Clustering score	−0.303 (0.07)	21.35	<0.0001	0.739 (0.650–0.840)
Constant	1.16 (1.29)	0.81	0.367	

B, beta coefficient; SE, standard error; Wald, Wald statistics; OR, odds ratio; CI, confidence interval.

We obtained age-, gender-, and education-adjusted MMSE (MMSE-T) using the equation as following from logistic regression models; *logit (case)  = 15.328 – 0.017× gender – 0.065× age +0.444× education level – 0.591× MMSE*. We also obtained age-, gender-, and education-adjusted MMSE (MMSE-T) using the equation as following; *logit (case)  = 2.460+0.552× gender – 0.019× age +0.243× education level – 0.289× CVFT total score*.

As shown in Table 3, the AUCs of MMSE-T (AUC_MMSE-T_) was greater than 0.90, indicating that the MMSE-T is useful for detecting AD. The AUC of the CVFT-T (AUC_CVFT-T_) was significantly smaller than the AUC_MMSE-T_ (difference  = 0.128, Standard error [SE]  = 0.015, 95% CI = 0.099–0.157, p<.001), indicating that the CVFT-T is much less accurate than the MMSE-T for diagnosis of AD. However, the AUC of the CVFT-C (AUC_CVFT-C_) was 0.903, which, although smaller than the AUC_MMSE-T_ (difference = 0.034, SE = 0.013, 95% CI = 0.010–0.059, p = .006), was much larger than the AUC_CVFT-T_ (difference = 0.094, SE = 0.013, 95% CI = 0.069–0.119, p<.001), indicating that the diagnostic accuracy for AD can be significantly improved by employing the CVFT-C instead of the CVFT-T.

**Table 3:** Diagnostic accuracies of the Mini Mental Status Examination (MMSE), Categorical Verbal Fluency Test total score (CVFT-T) and composite score (CVFT-C) for Alzheimer's disease.

	Cut- off^a^	Sensitivity	Specificity	AUC		
				AUC	SE	95% CI
MMSE	>0.5293	0.836 (0.798– 0.870)	0.893 (0.860– 0.921)	0.930	0.009	0.913– 0.946
CVFT-T	>0.4875	0.746 (0.702– 0.787)	0.751 (0.707– 0.792)	0.818	0.014	0.789– 0.846
CVFT-C	>0.6034	0.803 (0.762– 0.840)	0.816 (0.772– 0.854)	0.903	0.010	0.883– 0.923

^a^ Optimal cut-off scores for Alzheimer's disease by receiver operator curve (ROC) analyses from predicted probability of age-, gender-, and education-adjusted logistic regression model.

AUC, area under ROC; SE, standard error; 95% CI, 95% confidence interval.

The optimal cut-off scores for AD of the CVFT-C, CVFT-T, and MMSE-T were determined as 0.6034, 0.4875, and 0.5293, respectively. The sensitivity and specificity for AD of the MMSE-T were 0.836 and 0.893 respectively, at its optimal cut-off, in agreement with previously published data [23]. In the logistic regression analyses adjusting age, gender, and educational level, 90.2%, 81.8%, and 93.0% of diagnoses were correctly predicted by the CVFT-C, CVFT-T, and MMSE-T respectively, at their optimal cut-offs. The sensitivity and specificity of CVFT-C (sensitivity = 0.803, specificity = 0.816) were slightly lower than those of the MMSE-T, but much higher than those of the CVFT-T (sensitivity = 0.746, specificity = 0.751).

As shown in Table 4, the AUC_CVFT-C_ remained larger than 0.9 in the 100 boot-strapped resamples. We also found that the AUC_CVFT-C_ was much larger than the AUC_CVFT-T_ (difference = 0.085, SE = 0.011, 95% CI = 0.064–0.106, p<.0001) and the CVFT-C was more sensitive and specific than the CVFT-T in diagnosing AD.

**Table 4:** Bootstrap validation^a^ of the diagnostic accuracy for Alzheimer' disease of the Mini Mental Status Examination (MMSE), Categorical Fluency Test total score (CVFT-T) and composite score (CVFT-C).

	Cut-off	Sensitivity	Specificity	AUC		
				AUC	SE	95% CI
MMSE	>0.5293	0.835 (0.798– 0.872)	0.890 (0.856– 0.924)	0.930	0.008	0.915– 0.946
CVFT-T	>0.4875	0.746 (0.704– 0.789)	0.747 (0.704– 0.789)	0.816	0.014	0.789– 0.844
CVFT-C	>0.6034	0.803 (0.768– 0.837)	0.838 (0.800– 0.876)	0.901	0.011	0.880– 0.922

^a^ 100 runs; in each run, 60% samples were randomly selected for validations using bootstrap sampling estimation.

AUC, area under the receiver operator curver; SE, standard error; 95% CI, 95% confidence interval.

## Discussion

We developed a weighted composite score of the CVFT that was considerably more accurate in diagnosing AD than the conventional CVFT total score using quantitative and qualitative differences in performance on CVFT in AD patients and normal controls. We confirmed that the diagnostic accuracy of the composite score was much better than the CVFT total score via bootstrapping procedure using a large age- and gender-matched sample of community-dwelling AD patients and normal controls.

As previously reported, AD patients showed much lower total CVFT scores than the controls [20]. Both the first-half and second-half scores of the AD patients were lower than those of the controls. In addition to this quantitative difference, the AD group showed several qualitative differences in the performance of CVFT from the normal group. The AD group had fewer switching events than the control group, but the clustering, perseveration, and intrusion scores were comparable between the AD group and the control group. These observations agreed with previous reports in most [7], [24] but not all [25]. These inconsistent results may be attributable to several causes. First, the clustering score is partially reciprocal to the perseveration score, since perseveration errors were usually included in calculating clustering scores. Second, the clustering score may be also reciprocal to the switching score. In this study, 26% of AD patients did not switch subcategories at all and produced animal names in domestic animal subcategory only. Third, simultaneous reduction in both clustering and switching is more likely to happen as the severity of AD increases. We included only very mild-to-mild AD patients in the present study.

Although various methods could be applied to build predictive models for the clinical data with binary outcome variables, like the presence or absence of AD, we found that the multiple logistic regression method was a better choice in view of efficiency and accuracy [26]. Conventional screening of variable methods for logistic regression includes the forward-selection, backward-elimination, stepwise and best subset methods. The first 3 methods emphasize how to choose α, which is the cutoff at which the variables enter or are removed from the model. It is obvious that the α value is subjectively chosen, and a significance level for entry (SLENTRY) of 0.05 is too rigorous to allow inclusion of important variables from the model in some cases [27]. For possible combinations of variables, the best subset method can give the corresponding chi-square values but is not useful to help decide which type of combination is optimal [26]. Thus, we combined the best subset method with the Akaike Information Criterion (AIC) to screen variables quickly and easily. The method not only takes the performance of the model into account but also saves the “trouble” of artificially choosing of the α value. The criteria to assess the model fit are used by Akaike Information Criterion (AIC), Schwarz Criterion (SC), and −2 Log L. −2 Log L is negative 2 times the log likelihood. The −2 Log L is used in hypothesis tests for nested models. AIC is calculated as AIC  = −2 Log L +2 ([k-1] + s), where k is the number of levels of the dependent variable, and s is the number of predictors in the model. AIC is used for the comparison of models from different samples or non-nested models. Ultimately, the model with the smallest AIC is considered the best. We used the AIC criterion for model fit because the composite model is not a nested model of MMSE or CVFT total score.

Since the AUC_CVFT-C_ was larger than 0.9, the CVFT can be a good screening instrument if we use the CVFT-C instead of the CVFT-T. Although the AUC_CVFT-C_ was slightly lower than the AUC_MMSE-T_, the CVFT has several strengths as a promising alternative to the MMSE as a screening instrument for AD. It is much briefer to administer, easier to administer, and more widely applicable than the MMSE. Furthermore, it can be far more accessible than the MMSE since its administration can be easily standardized and automated. Since it can be easily administered over the phone or internet, it may help overcome regional inequality of getting AD screen due to the regional difference in the health resources and lower the screening cost for AD. Recently we developed and posted an Android app for screening AD using the CVFT-C named ‘The Dementia Traffic Light’ (https://play.google. com/store/apps details?id = com.sunbh.dementia). Since several recent studies showed the feasibility of testing cognition over the phone, [28], [29] we may also develop a telephone version of ‘The Dementia Traffic Light’ in the future. The target of these programs may be primarily mild AD and MCI patients since these programs may be difficult to carry correctly for moderate to severe AD patients.

The current study has several strengths. First, we validated the results with bootstrap procedures using an age- and gender-matched sample. This validation offered statistical confidence in the generalizability of our observations. Second, we included only very mild–to-mild AD patients in the current study. A number of previous studies aimed at developing screening instruments for AD included severe AD patients, which might have resulted in overestimation of the diagnostic accuracy of the tools described. Confining test subjects to mild cases may be more useful for evaluating screening tests. Third, the current study was performed with a well-powered sample size. Fourth, both AD patients and normal controls took a standardized structured diagnostic assessment for AD.

**Two limitations of the present study also require consideration. First, the control group did not include cognitively impaired but not demented elders with CDRs of 0.5. This may have exaggerated the reported diagnostic accuracies. Second, because of the cross-sectional nature of the present study, a prospective longitudinal study is warranted to determine whether the CVFT-C may predict the risk of AD in the elderly.**
